# Zero Waste Concept in Production of PLA Biocomposites Reinforced with Fibers Derived from Wild Plant (*Spartium junceum* L.) and Energy Crop (*Sida hermaphrodita* (L.) Rusby)

**DOI:** 10.3390/polym17020235

**Published:** 2025-01-18

**Authors:** Zorana Kovačević, Ana Pilipović, Mario Meheš, Sandra Bischof

**Affiliations:** 1 Faculty of Textile Technology, University of Zagreb, Prilaz baruna Filipovića 28 a, 10000 Zagreb, Croatia; 2 Faculty of Mechanical Engineering and Naval Architecture, University of Zagreb, Ivana Lučića 5, 10002 Zagreb, Croatia; ana.pilipovic@fsb.unizg.hr; 3 Faculty of Chemical Engineering and Technology, University of Zagreb, Trg Marka Marulića 19, 10000 Zagreb, Croatia; mmehes@fkit.hr

**Keywords:** biocomposites, mechanical properties, thermal properties, nanoclay, polylactide, *Sida hermaphrodita* (L.) Rusby, *Spartium junceum* L.

## Abstract

This research follows the principles of circular economy through the zero waste concept and cascade approach performed in two steps. Our paper focuses on the first step and explores the characteristics of developed biocomposite materials made from a biodegradable poly(lactic acid) polymer (PLA) reinforced with natural fibers isolated from the second generation of biomass (agricultural biomass and weeds). Two plants, *Spartium junceum* L. (SJL) and *Sida hermaphrodita* (SH), were applied. To enhance their mechanical, thermal, and antimicrobial properties, their modification was performed with environmentally friendly additives—linseed oil (LO), organo-modified montmorillonite nanoclay (MMT), milled cork (MC), and zinc oxide (ZnO). The results revealed that SH fibers exhibited 38.92% higher tensile strength than SJL fibers. Composites reinforced with SH fibers modified only with LO displayed a 27.33% increase in tensile strength compared to neat PLA. The addition of LO improved the thermal stability of both biocomposites by approximately 5–7 °C. Furthermore, the inclusion of MMT filler significantly reduced the flammability, lowering the heat release rate to 30.25%, and enabling the categorization of developed biocomposite in a group of flame retardants. In the second step, all waste streams generated during the fibers extraction process are repurposed into the production of solid biofuels (pellets, briquettes) or biogas (bio)methane.

## 1. Introduction

The era of development of composites with a synthetic polymer matrix reinforced with synthetic fibers began already 100 years ago [1]. This type of composite was represented in a wide range of industries due to its good properties, such as high strength and stiffness, enhanced dimensional stability, and thermal properties [2]. In recent years, due to increasing environmental concerns, the development of green composite materials whose matrix and reinforcement are both made from renewable and biodegradable sources, has been initiated. One of the most commonly used thermoplastic and biodegradable polymers is poly(lactic acid) (PLA). It is one of the most studied polymers of the aliphatic polyester family because it is produced via the fermentation of renewable resources, like sugar, beets, or corn starch [3]. Natural bast fibers are most commonly used as reinforcement in green biocomposites due to their favorable properties of wide availability, low price, and good mechanical properties [4,5]. The good mechanical properties of natural plant fibers (bast fibers) are due to cellulose—a natural polymer with a chain structure that is the main component of plant cell walls. One possible source of cellulose fibers is agro waste. Agro waste is a lignocellulosic material by its chemical composition, which, in addition to lignin and hemicellulose, is abundant in cellulose-rich areas. Most often, agro waste in the form of stems is thrown away, burned or buried in the ground, which leads to environmental pollution instead of being used as a source of cellulose fibers that can subsequently be used as reinforcements for composite materials. Due to the continuous increase in the global population, enhanced demand for food, and care for the preservation of agricultural land, novel procedures for isolating fibers from agricultural biomass waste have been developed [2].

**Figure 1:**
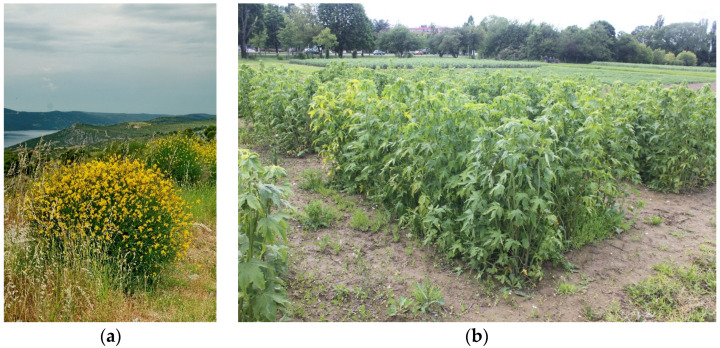
(**a**) Spanish broom (*Spartium junceum* L.) and (**b**) Virginia mallow (*Sida hermaphrodita* (L.) Rusby).

In this work, fibers from two different plants, Spanish broom (*Spartium junceum* L.) and Virginia mallow (*Sida hermaphrodita* (L.) Rusby) were isolated for biocomposite reinforcement (Figure 1).

*Spartium junceum* L. is a wild plant from the leguminous family (*Fabaceae*/*Luguminosae*) that mostly grows in Mediterranean countries. Throughout history, it has had a wide range of different applications (e.g., for scents and dyes derived from flowers, baskets made from stems, and textile materials made from isolated fibers). Fibers are the most valuable product, so there has been a renewed interest in their production in recent times [3,6,7,8,9].

*Sida hermaphrodita* belongs to the mallow family (*Malvaceae*) and is native to the USA and Canada [10]. In the 1930s, its cultivation began in Poland primarily as a fodder and fiber crop [11], but when its favorable energetic properties were discovered, cultivation for energy purposes began [12,13,14]. In order to meet the high demand for biomass and avoid the use of soils suitable for food production, the future cultivation of energy crops must rely on marginal and poor-quality soils. One such energy plantations that thrive on unfavorable soils is the *Sida hermaphrodita* one, which enables the production of biofuel with an exploitation time of up to 20 years [10]. Cultivation of *Sida hermaphrodita* does not require any special soil conditions. However, to achieve a high biomass yield, there is still a need for moderate fertilization. Nabel, M. et al. (2018) suggested intercropping of *Sida hermaphrodita* with legumes, which have the ability to fix nitrogen (N) from the atmosphere and enrich the cropping system with this essential nutrient and consequently increase the biomass yield [15]. The cultivation of *Spartium junceum* L., which is considered a weed, in combination with *Sida hermaphrodita* on poor-quality soils is one of the ideas that will be examined in our future research. Taking into consideration that the fibers of *Spartium junceum* L. are already categorized as natural bast fibers, maceration methods characteristic for flax fibers isolation were applied [16,17]. The fibers were isolated in the same way and characterized to determine the possibility of their application for biocomposites. The process of isolating fibers in a low-concentration alkaline medium using microwave energy showed promising results, both from the ecological and economic side. The advantages of natural fiber reinforced composites (NFRC) lie in their low mass, density, and price, combined with high strength. However, it is equally important to emphasize the negative aspects related to the interaction of hydrophilic natural fibers with a hydrophobic polymer matrix. Since natural fibers do not show good compatibility with non-polar polymer matrices, it is necessary to modify the fibers or the matrix to achieve better adhesion between these layers [18,19]. Therefore, it is often necessary to add fillers to enable composite usage in highly demanding applications.

Although PLA is one of the most used polymers in biocomposites due to its good mechanical properties [20,21], they can be further enhanced with the addition of drying vegetable oils (i.e., linseed oil) as plasticizers [22]. Plasticizers affect the processability of PLA by lowering the melt viscosity and increasing its ductility [23]. The requirement to substitute conventional with sustainable materials and retain superior properties is derived development of bio-based fillers obtained from renewable sources or industrial by-products [24]. This research combines conventional fillers, such as metal oxide and nanoclay, with renewable and sustainable ones, such as cork. Zinc oxide (ZnO) fillers have already proven their antimicrobial activity, non-toxicity, stability, availability, and low cost. In this paper, micro-sized ZnO particles were used since European regulation considered nanosized ZnO particles as potentially nanotoxic, while microparticles of ZnO show adequate antimicrobial properties with low cytotoxicity [25]. Furthermore, Mitjans, M. et al. (2023) pointed out that no acute toxicity was observed at low ZnO concentrations [26]. Venkatesh, C. et al. (2020) have coated PLA with ZnO microparticles and tested its biodegradability in biological fluids—the simulated body fluid and artificial urine. Complete dissolution of the ZnO layer was observed after a 5-day treatment for the samples immersed in artificial urine [27]. ZnO has been used as a filler in composites in order to improve antimicrobial properties and combined with nanoclay to improve the flame retardancy of the final product. Nanoclays are commonly used as reinforcing fillers for the improvement of composite system thermal stability. These layer silicates are applied due to their availability, versatility, and respectability towards the environment and health [3]. The silicate layers are miscible only with hydrophilic polymers, while for hydrophobic polymers, organic compounds have to be introduced. Such organic modification improves not only compatibilization between hydrophilic clay and hydrophobic polymer matrix but also increases interlayer spacing as well [3]. Additionally, natural cork was added since it exhibits good compressibility, flexibility, durability, acoustic insulation, and improved flame retardancy and, therefore, has broad application in the automotive, construction, food, and aviation sectors [28,29]. Cork is the outer bark of the oak tree with a pronounced alveolar structure that reminds of honeycomb [30]. Fabijanski, M. (2024) combined PLA and cork filler in quantities ranging from 5% to 30% and its positive influence on hardness and decrease in the formation of agglomerates inspired us to perform further investigations [28].

### 1.1. State of the Art on Spartium junceum L. Reinforced Composites

Composites reinforced with SJL fibers are relatively new products. An Algerian group of scientists introduced them in 2006. Nekkaa, S. et al. (2006, 2009, 2012) developed *Spartium junceum* fiber-reinforced polypropylene (PP) composites. The weight fraction of used fibers inside the composite was 10–50 wt.% and fibers were cut to the length ranging from 2 mm to 4 mm. They have found that composites reinforced with silane-treated fibers show an increase in composite stiffness and a loss in bending modulus compared to neat PP. Silane treatment reduces the water absorption capacity of composites while impact properties of such water-saturated composite samples were very poor [31,32,33]. Kovačević, Z. et al. (2012, 2014, 2015, 2018, 2019) developed composites made of PLA polymer and 20 wt.% of SJL fibers, previously cut to the length of 2–5 mm. Prior to the composite production, the fibers were modified with nanoclay and citric acid for the improvement of mechanical and thermal properties. Significant improvement in strength (164%) was obtained in comparison to the neat PLA polymer [34,35,36,37,38]. Chidichimo, G. et al. (2015) have used a milled SJL plant (approx. 50 μm granulation) ranging from 5 wt.% to 30 wt.% as reinforcement of polyurethane matrix. Improved mechanical properties were obtained in the presence of minimally 20 wt.% of SJL fibers [39]. Bouhank, S. et al. (2016) analyzed water absorption characteristics, thermal degradation, and morphological properties in their study of SJL-reinforced poly(vinyl chloride) composites. The applied fiber size ranged from 200 to 400 μm. Water uptake of composites increased with the fiber content increase. Therefore, fiber modification with alkali and silane was needed for the reduction of water uptake and increase in composite impact properties [40]. Bedreddine, M. et al. (2019) added SJL flour into the PLA matrix. The disappearance of peaks at 1740 and 1250 cm^−1^ indicates that alkali treatment has removed hemicellulose and a part of lignin. The bands between 1420 and 1430 cm^−1^ and at 900 cm^−1^ are assigned to the contributions of the crystalline and amorphous structures into the cellulosic fibers. Melting endotherm temperature is increasing at 7 °C from neat polymer to composite indicating that in the presence of SJL fibers, more ordered crystals are produced due to the fiber’s nucleating effect. The melting enthalpies depend on the amount of PLA in the composite material [41]. Messaoudi, K. et al. (2019) have used SJL flour as reinforcement of polypropylene composites. SJL flour was treated with maleic anhydride and silane and ester linkages were formed due to the reaction between maleic anhydride and SJL hydroxyl groups. Applied chemicals influence the increase in the SJL crystallinity index because of the removal of amorphous material that covers the fiber. Additionally, the incorporation of SJL flour in the polypropylene matrix increases the composite crystallinity due to the heterogeneous nucleation of the PP matrix. SJL flour reinforcement significantly increases the composite resilience and decreases composite water uptake. An increase in crystalline temperature Tc indicates that SJL flour acts as a nucleating agent and favors the polypropylene phase crystallization within the composites by increasing the crystallization rate. FTIR results show that chemical treatment of SJL fibers shifted the band at 1725 cm^−1^ (carbonyl groups) to a lower wavelength and induced a significant increase in the carbonyl groups intensity, which confirmed the formation of ester groups. Higher flour content directly decreases composite impact strength [42]. Nouar, Y. et al. (2020) applied SJL flour (with an average particle size below 100 µm) as reinforcement of polypropylene matrix in biocomposites. In order to enhance the interfacial interactions between fibers and matrix, authors have modified the fibers with 2 wt.% of sodium hydroxide (NaOH) and 5 wt.% of vinyltrimethoxysilane (VTMS). Alkali treatment increased the relative crystallinity of SJL flour particles while silane treatment had a minor influence on the crystallinity degree. SJL flour content above the 20 wt.% caused a significant increase in composite thermal conductivity leading to poor insulating properties [43]. Corapi, A. et al. (2023) investigated the potential toxicity of polyurethane biocomposites reinforced with functionalized SJL fibers. The addition of SJL fibers in polyurethane matrix shows positive ecotoxicological effects on the aquatic and atmospheric environments since cellulose fibers pose a considerable amount of chemical functional groups that immobilize free organic ammines and amides presented in polyurethane structure that are released in leachates [44]. Govorčin Bajsić, E. et al. (2023) investigate the impact of SJL fiber content on the thermal properties of different composite materials with polypropylene, polycarbonate, and thermoplastic polyurethane (TPU) matrix. A decrease in crystallinity degree was observed in comparison to the neat polypropylene matrix. The thermal stability of the composites with polypropylene and polyurethane matrix increased while in the composites with polycarbonate matrix it decreased with the fiber content increase [45]. Juradin, S. et al. (2023) used SJL fibers as reinforcement in cement mortar. They applied fibers of 1 cm and 3 cm length at 0.5 vol.% content and obtained an increase in flexural and compressive strength of 13.5% and 11.7%, respectively. Therefore, SJL fibers already found their way to the construction industry as reinforcement in cement constructions [46].

### 1.2. State of the Art on Sida hermaphrodita (L.) Rusby Reinforced Composites

*Sida hermaphrodita* (L.) Rusby (SH) has not been applied as reinforcement of composites up to now. This plant was initially used to produce fibers until its excellent energy properties were revealed. Nowadays, it is most represented as a raw material in the production of biofuels [47]. Czarnecki, R. et al. (2010) have applied pulverized stem of SH plant and various kinds of resin for particle board production. Results revealed that low-density boards made with the addition of SH are not very different from those made solely from wood particles [48]. Furthermore, SH fibers were applied in the construction industry as reinforcement of cement constructions. Khadka, R. (2021) developed eco-friendly concrete building material reinforced with SH stems of 0.5–2 cm in length. According to their results of density, compressive and flexural strength, and thermal conductivity, concrete reinforced with SH shows sufficiently good properties and confirms its application in the construction field [49]. A few studies were recently performed to develop more sustainable raw materials for the pulp and paper industry. Holler, M. et al. (2021) confirmed that SH could become a profound alternative to common energy and fiber plants. They revealed good paper properties and confirmed usage for one- or multilayer cardboard packaging where fiber strength is needed [50]. Kmiotek, M. et al. (2024) have studied the SH plant as a non-woody raw material for papermaking. Its chemical composition and morphological characteristics designated these fibers as suitable for various paper production (e.g., for printing, writing, and tissue paper) [51].

The aim of this paper was to develop biocomposite material reinforced with long natural fibers derived from the wild plant *Spartium junceum* L. and energy crop *Sida hermaphrodita* and to drive its functionalization towards the automotive industry. The previously proven positive influence of various additives for the improvement of composite mechanical, thermal, and antibacterial properties guided our choice, but the applied combination of MMT, ZnO, and cork presents novelty, and results are summarized in patent pending [52].

## 2. Materials and Methods

### 2.1. Materials

SJL fibers were isolated from plants harvested from the area around town of Šibenik, Croatia. SH fibers were isolated from plants and harvested from the experimental field of the University of Zagreb Faculty of Agriculture situated near the park Maksimir, Zagreb, Croatia. Sodium hydroxide pellets (NaOH), purity ≥ 97%, and nanoclay modified with 25–30 wt.% octadecylamine were obtained from Sigma-Aldrich Inc., Dorset, UK. Zinc oxide (ZnO), p.a. purity ≥ 99% and particle size < 5 µm, was obtained from Kemika d.d., Zagreb, Croatia. PLA polymer 6201D was purchased from Nature Works LLC, Plymouth, USA (specific gravity 1.24, relative viscosity 3.1, melt index 15–30 g/10 min, melt density 1.08 g/cm^3^). Milled cork (MC) was obtained by milling wine bottle stoppers in a cryogenic mill 6875, SPEXSamplePrep LLC, Metuchen, NJ, USA, through five cycles for 15 min in total.

### 2.2. Methods

#### 2.2.1. Fibers Isolation

Both fibers (SJL and SH) were extracted from the plant according to the method described in Kovačević, Z. et al. (2015) [36], with the acceptance that the processing time was increased for SH plants from 5 min to 20 min.

Fiber isolation was performed in a domestic microwave oven YC-GG252A, Sharp, Osaka, Japan, under 900 W and 2.45 GHz frequency. Plant’s stems were treated in PTFE round container ⌀ 16.5 cm. Treatment was conducted by using 5 wt.% NaOH and solid-to-liquid ratio was 1:6. After microwave treatment stems were washed in hot and cold water, neutralized with 1.5% acetic acid (CH_3_COOH), and finally rinsed with distilled water to obtain pH 7.

#### 2.2.2. Fibers Moisture Content and Moisture Regain

The moisture regain and content were calculated according to ASTM D2495-07 [53]. After the sample was air-dried and weighed, it was placed in a climatic chamber and exposed to standard atmospheric conditions for 24 h. Afterwards, the sample was weighed again and subjected to another cycle of drying for 24 h. The moisture regain and content were calculated according to Equations (1) and (2). Testing was carried out in triplicate to ensure precision and consistency.

(1) $$MC\%=\left(\frac{m_{1}-m_{2}}{m_{1}}\right)\times 100$$

(2) $$MR\%=\left(\frac{m_{3}-m_{2}}{m_{2}}\right)\times 100$$

where *MC*% represents moisture content, *MR*% represents the moisture regain, *m*_1_ (g) represents the mass of an air-dried sample, *m*_2_ (g) represents the completely dried sample’s mass, and *m*_3_ (g) represents the conditioned sample’s mass.

#### 2.2.3. Fiber Density

The sample density was evaluated with Ultrapyc 1200e, Anton Paar, Boynton Beach, FL, USA, gas pycnometer. The density of the fibers was conducted according to ASTM D8171-18 method [54]. Nitrogen gas (N_2_) of high purity was utilized due to its capability to seep into the minuscule pores, thus augmenting the precision of the measurement. The measurements were performed in triplicate to guarantee accuracy.

#### 2.2.4. Fibers Mechanical Properties

Mechanical properties of individual fibers were measured using the Vibroskop 500 and Vibrodyn 500, Lenzing Instruments, Gampern, Austria. Preload, testing speed, and gauge length were 1500 mg, 3 mm/min, and 5 mm, respectively. Samples were adjusted to standard conditions (temperature and humidity) prior to the examination. An average of 100 measurements for individual fibers was used in this study.

#### 2.2.5. Fiber Morphology

Morphological features of plant stem cross-section, fiber cross-section, and fiber longitudinal view were measured by scanning electron microscope FE-SEM Mira II LMU, Tescan, Brno, Czech Republic. Prior to SEM examination, samples for plant cross-section analysis were prepared by immersing them into Dewar filled with liquid nitrogen. Afterward, they were cut and sputter-coated with Cr in order to increase their electrical conductivity. SEM imaging was conducted at 5.00 kV voltage and the following magnifications were taken: 333×, 2.00k×, and 6.68k×.

#### 2.2.6. Composite Formation

PLA pellets were used to prepare sheets in vacuum oven VO 49, Memmert, Nuremberg, Germany, at 180 °C, under 700 mbar pressure for 5 min. All composite constituents (fibers, PLA, MMT, ZnO, MC) were absolutely dried at 100 °C for 24 h prior to the processing. Slurries made of linseed oil 10 wt.%, MMT 1 wt.%, ZnO 1 wt.%, and MC 1 wt.% were prepared by using laboratory Vortex Mixer MX-S, DLAB until the homogeneity has been achieved. Then, 20 wt.% of fibers were combed and parallelized before the treatment with the slurry. Treated fibers were manually laid in one direction between two PLA sheets and this sandwiched structure was placed inside the square aluminum mold of 80 cm × 80 mm × 3 mm. Such assembly was pressed in the laboratory compression molding press LAB 100 C, Pinette PEI at 190 °C and 30 kN pressure for 3 min with a 30 s degassing cycle.

Table 1 shows a legend of the labels of each specimen made in this experiment.

After composite preparation, test specimens were prepared by cutting them to desired size of 10 mm × 80 mm × 3 mm on the laser cutting machine Fabcore FC21-MK3, FabCreator, Mierlo, Netherlands, for further analysis.

#### 2.2.7. Composite Mechanical Properties

The tensile properties were determined according to the HRN EN ISO 527-5:2021 [55] using a universal testing machine UTM 1445, Zwick, Ulm, Germany, with a force of 10 kN. The test was conducted at room temperature with a testing speed of 3 mm/min. The gauge length was 50 mm. The tensile tests were carried out in five repetitions for each sample, and the average results and standard deviation were subsequently calculated. During the test, force and elongation of the specimen, stress, strain, and Young’s modulus were measured.

The flexural properties testing was conducted in accordance with the standard HRN EN ISO 14125:2005 [56] using a AGS-X Shimadzu, Tokyo, Japan, device with a maximum force of 10 kN. The test was conducted at room temperature with a testing speed of 2 mm/min. The distance between the spans was L = 48 mm. The flexural test was conducted on three specimens for each condition (mixtures of different components), as well as on three specimens of pure PLA. The average results and standard deviations were calculated. During the test, force and elongation of the specimen, stress, strain, deflection, and flexural modulus were measured.

Impact strength testing was conducted according to the standard HRN EN ISO 179-1:2023 [57] using a Charpy pendulum, Karl Frank GmbH, Weinheim-Birkenau, Germany at room temperature. The nominal pendulum value was 2 J, and the support span was 62 mm. The same testing methodology was employed for impact strength assessment, utilizing five specimens for each condition alongside five specimens of pure PLA, with averages and standard deviation calculated afterward.

#### 2.2.8. Composites Thermal Properties

Thermogravimetric analysis (TGA) was studied using a TGA/DSC3+, Mettler-Toledo, Greifensee, Switzerland. The 10 mg amount of samples were put in ceramic pan of 60 μL volume and heated from 25 °C to 600 °C at 10 °C/min heating rate in a nitrogen gas flow (60 mL·min^−1^, purity 99.999%).

Differential scanning calorimetry (DSC) analysis was studied using a DSC 822e, Mettler-Toledo, Greifensee, Switzerland, device with purged dry nitrogen gas flow (40 mL·min^−1^, purity 99.999%), previously calibrated with indium and zinc. About 10 mg of samples were encapsulated in standard aluminum pans with pierced lids. The following thermal cycles were applied: (1) dynamic heating from 25 to 200 °C at 10 °C/min, (samples were held at this temperature for 3 min to erase the processing and thermal history), (2) dynamic cooling from 200 to 0 °C at 10 °C/min and held at 20 °C for 3 min, (3) reheated to 200 °C at 10 °C. The cooling was completed under liquid nitrogen. Glass transition temperature (Tg), cold crystallization (Tcc), melting temperature (Tm), and crystallization temperatures (Tc) were obtained from the endothermic and exothermic peaks in the heating and cooling scans, respectively. Degree of crystallinity (*χ_c_*) of composites was calculated using Equation (3):

(3) $$\chi_{c}=\left(\frac{∆H_{m}-∆H_{cc}}{{{∆H}_{m}}^{0}\times w_{PLA}}\right)\times 100$$

where ∆*H_m_* is the enthalpy of melting (J·g^−1^) determined by the DSC measurement, ∆*H_cc_* is the cold crystallization enthalpy and ∆*H_m_*^0^ is the melting enthalpy value of the purely crystalline PLA (%), *w_PLA_* (g) is the mass content of PLA. The value of melting enthalpy of the pure PLA crystals is 93 J/g.

Microscale combustion calorimetry (MCC) was used to investigate the heat of combustion of gases evolved during the controlled heating of the specimens. The analysis has been conducted on MCC-2, Concept Equipment, West Sussex, UK, device. The heat release rate (HRR) curves, as well as corresponding combustion data, were calculated based on three replicate measurements according to ASTM D7309-21a (Method A) [58].

## 3. Results and Discussion

### 3.1. Fibers Physical Properties

**Figure 2:**
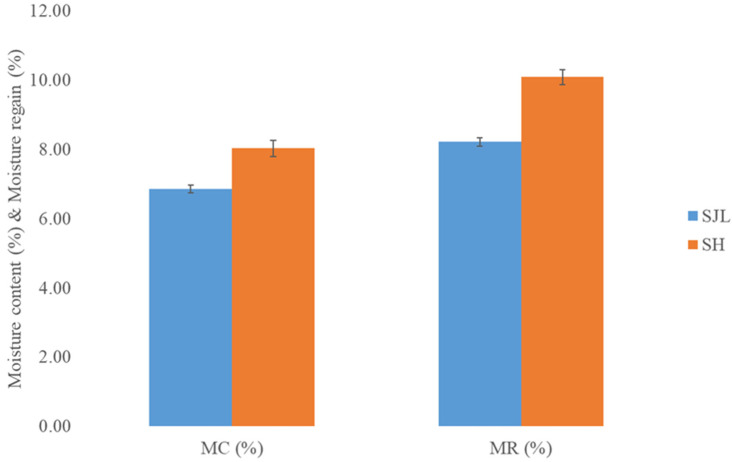
Moisture content (MC) and moisture regain (MR) of *Spartium junceum* L. (SJL) and *Sida hermaphrodita* (SH) fibers. Error bars represent 95% confidence interval.

The moisture content and regain of the SJL and SH fibers are presented in Figure 2. The fibers were isolated from the plant stems a few months after the harvest period; therefore, the moisture measurements were obtained at the same time for all the investigated fibers. Within the period from isolation to moisture measurements, fibers were stored in sealed and clean polyethylene bags in the normal indoor atmosphere.

Fibers isolated from *Sida hermaphrodita* exhibit higher moisture content and moisture regain values (8.03% and 10.09%, respectively) compared to SJL fibers (6.86% and 8.22%, respectively). The difference between both moisture content and moisture regain mean values is noticeable due to the fact that F_critical_ is lower than F_statistical_, while the *p*-values are lower than 0.05. Relatively low moisture content in SJL fibers is often considered acceptable for many textile applications, especially for the storage, transportation, and processing of isolated fibers. It is important to note that the optimal moisture content for natural fibers can vary depending on the intended application and industry standards. Additionally, the moisture content of fibers can change over time, depending on the environmental conditions. Proper storage and handling are essential to maintaining the desired moisture levels in natural fibers for various applications, especially for composite production [59,60,61].

**Figure 3:**
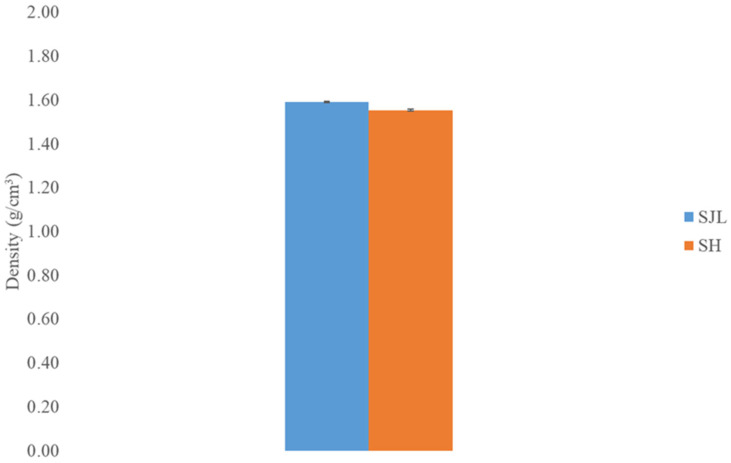
Density of *Spartium junceum* L. (SJL) and *Sida hermaphrodita* (SH) fibers. Error bars represent 95% confidence interval.

Fiber density results are presented in Figure 3. Measured values are in accordance with the density values of other bast natural fibers, i.e., flax fibers [52,62].

The conditions in which plants are grown and harvested can influence fiber density. Factors like soil quality, climate, and agricultural practices can affect the fiber’s overall structure and, consequently, its density. Since natural fibers show great variability in their properties, factors of fiber diameter, wall thickness, and internal structure can vary, leading to differences in their density [63]. SH fibers show significantly lower density compared to SJL fibers (1.55 g/cm^3^ and 1.59 g/cm^3^, respectively). Following the one-way ANOVA analysis, a statistically noticeable difference in density was observed between these fibers (*p*-value < 0.05). One of the key demands in composite materials design is their lightweight combined with the retention of their strength. This is the reason why natural fibers are of huge interest as reinforcing material for composites. Natural fiber’s addition to the polymer matrix influences the formation of voids and the presence of higher air content while its low density affects the density of composite material and makes it lightweight [64].

### 3.2. Fibers Mechanical Properties

Results of breaking tenacity, elongation, and fineness of individual fibers are presented in Table 2.

SH fibers show an increase in fiber tenacity and modulus of 38.92% and 24.05% compared to SJL fibers, indicating their higher flexibility [65]. According to our results, SH fibers are stiffer than SJL fibers and their value of Young modulus is in the same range as sisal (9.4–22 GPa), ramie (24.5 GPa), or jute (26.5 GPa) [66]. Young’s moduli of both fibers are relatively low in comparison to flax (15–54 GPa) and hemp (17–70 GPa) fibers [65,67]. Fibers’ mechanical properties such as Young’s moduli and strength are influenced by microfibril angle, chemical composition, irregular cell geometry, secondary cell wall, fiber cross-sectional area, and lumen size [66,68,69]. Bast fibers have a lumen, a central channel that is situated in the middle of the fiber, which usually is not subtracted from the fiber cross-section. The discrepancy of Young’s moduli results is affected by the size of the cross-sectional area. Therefore, Kempe, A. et al. (2015) in their research based on *Carica papaya* L. bast fibers concluded that calculated Young’s modulus and strength are lower than they are in reality [65]. The strength of SH fibers is 1240.38 MPa, which is considerably higher than the values of other bast fibers: ramie (400–938 MPa), jute (400–800 MPa), kenaf (284–800 MPa), flax (800–1500 MPa) and hemp (550–900 MPa) [70,71]. Therefore, this characteristic makes SH an excellent candidate for reinforcement of biocomposite production.

### 3.3. Fiber Morphology

**Figure 4:**
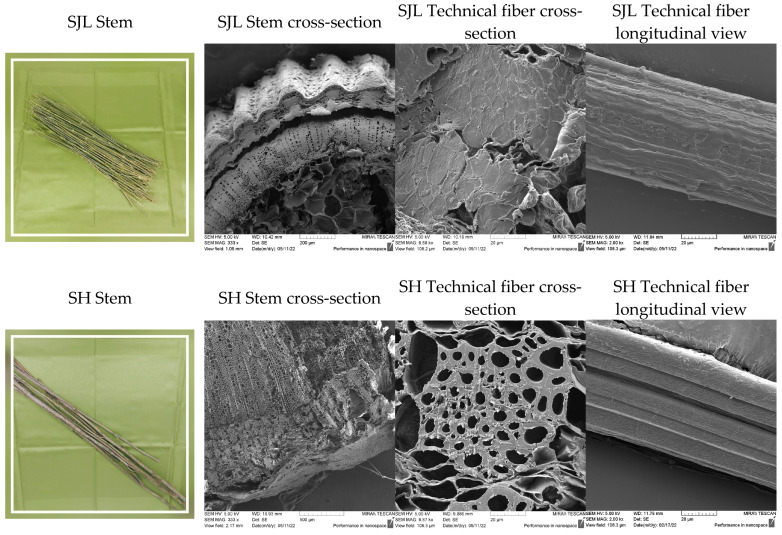
SEM micrographs taken under the following magnifications—333×, 6.68k×, and 2.00k×. First row: *Spartium junceum* L. stem and technical fiber morphology. Second row: *Sida hermaphrodita* stem and technical fiber morphology.

The morphology features of SJL and SH fibers are presented in Figure 4. The cross-sectional area of the stem consists of similar layers in both plants: xylem, phloem, sclerenchyma (bast fibers), and epidermis [3]. Bast fibers are located in the outer part of the stem. The fibers called technical fibers are bundles of elementary fibers whose width ranges from 10 to 20 µm (Figure 4: last micrograph in each row). Surface features within the longitudinal view of both tested fibers are rather similar although SH fibers show a more uniform appearance in terms of surface irregularities. SEM analysis of the fiber surface shows wrinkles, grooves, and kink bands, which are characteristic of bast fiber topography. A cross-section of technical fibers presents irregular shapes of elementary fibers situated within the bundle. SJL fibers show a thick secondary cell wall and small-sized lumen, while SH fibers show considerably larger lumen. Rough surface and large lumen position SH fibers in the category of fibers suitable for reinforcement in polymer composite materials due to mechanical interlocking between fibers and polymer. Fiber type (category) is a very important indicator of the fiber’s further performance. Bazli, L. et al. (2021) pointed out that bast fibers have high flexural strength while leaf fibers show favorable impact properties [72].

### 3.4. Composites Mechanical Properties

The mechanical properties of composite materials were investigated with tensile, flexural, and impact properties. These properties are influenced by the fiber’s tensile strength and modulus, adhesion between the matrix and fibers, impact resistance, and others [73,74]. The addition of fillers to NFRC significantly influences their mechanical properties. Some of the key mechanisms by which fillers affect these properties are listed in Table 3.

The overall effect of fillers on the mechanical properties of NFRC depends on several factors, including the type, size, shape, and concentration of the fillers, as well as the processing conditions and the nature of the matrix and fibers [75]. Therefore, in this paper, the positive aspects of the filler on the mechanical properties were not fully confirmed due to the low concentration of the filler.

**Figure 5:**
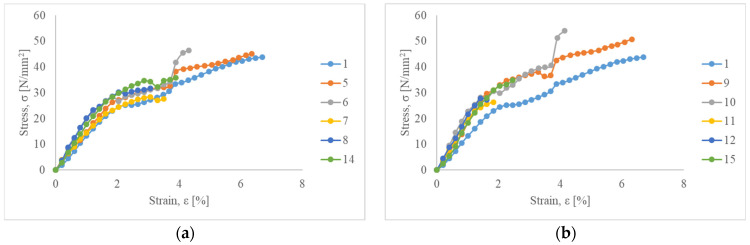
Tensile stress–strain behavior of neat PLA compared to (**a**) SJL fiber-reinforced composites; (**b**) SH fiber-reinforced composites.

Figure 5 presents the tensile stress–strain behavior of natural fiber-reinforced composites compared to neat PLA (polymer matrix). It is noticeable that composites without various fillers such as LO, MMT, ZnO, and MC break at a higher strain, beyond 6% (Specimens 5 and 9). Composites reinforced with fibers modified with LO show the highest strength values of 46.51 MPa for Specimen 6 and 54.37 MPa for Specimen 10.

Neat PLA (Specimen 1) shows tensile strength and modulus of 44.35 MPa and 0.89 GPa, respectively (Table 4). Its strain at break is 6.45%. The addition of linseed oil (Specimen 6 and Specimen 10) improved tensile strength and modulus, which enhances stiffness, meaning that the composite can bear more load while undergoing less deformation.

Other specimens show a decrease in Young’s modulus indicating that a combination of LO and other fillers could induce a plasticizing effect and thus better flexibility of such specimens [76]. According to Orue, A. et al. (2018), a combination of epoxidized vegetable oils and fibers could improve the strain at break and tensile modulus by 70% and 30%, respectively [77]. Composites with incorporated fillers such as MMT, ZnO, and MC show a negative impact on the tensile strength of natural fiber-reinforced composites (Specimens 7, 8, 14, 11, 12, and 15). Introduction of 20 wt.% natural fibers (SJL and SH) to polymer matrix without additional fillers influences better tensile properties. Composites reinforced with SJL (Specimen 5) and SJL fibers modified with LO (Specimen 6) show an increase in strength of 2.3% and 4.9%, respectively, compared to neat PLA. Composites reinforced with SH (Specimen 9) and SH fibers modified with LO (Specimen 10) show a higher increase in strength of 14.36% and 27.33%, respectively, compared to neat PLA. Better results of SH-reinforced composites are in line with their better tensile properties in comparison to SJL fibers. Additionally, the lumen size of SH fibers is significantly larger than SJL fiber as can be noticed in Figure 4. Yusoff, R. B. et al. (2016) in their research pointed out that the size of the fiber lumen is very important since the penetration of the matrix into the lumen could create better interlocking behavior regarding cohesion in the cell wall so loads could be withstood and less fiber pull-out could be noticed [68].

**Figure 6:**
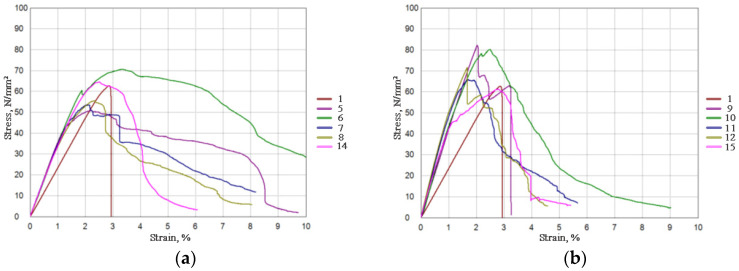
Flexural stress–strain behavior of neat PLA compared to (**a**) SJL fiber-reinforced composites; (**b**) SH fiber-reinforced composites.

The flexural properties of investigated fiber-reinforced composite materials are presented in Table 5 and Figure 6.

The flexural strength and modulus of neat PLA were 62.83 MPa and 2.35 GPa, respectively. Composites containing ZnO show decrement in flexural strength for composites reinforced with SJL fibers (Specimens 7 and 8). Composites reinforced with SH fibers show an increase in flexural strength and modulus for all SH-reinforced composites, but the increase in flexural strength is less pronounced on specimens that contain ZnO. Figure 6 presents average values of measured stress–strain behavior where the modulus of elasticity in bending (flexural modulus) is depicted by the slope of the stress–strain curve. SH-reinforced composites exhibit a steeper linear slope of the stress–strain curves indicating a higher modulus of elasticity. An increase in flexural modulus and therefore an increase in material stiffness (resistance to bend) is observed for all of the tested specimens (SJL and SH composites) compared to neat PLA, which is influenced by the addition of various fillers [78].

During the analysis of flexural properties, the test specimen is exposed to compression and tension stress. Therefore, stiffness is an important property since it protects the composite system from deflecting [79,80]. Ochi, S. (2015) revealed the relationship between flexural strength and modulus with fiber content since the flexural strength and modulus increase linearly with the increase in fiber content. Composites with a volume fraction of bamboo fibers of 70% show a noticeably higher flexural strength and modulus of 273 MPa and 6.8 GPa, respectively [81]. In their research, Mazur, K. E. et al. (2022) concluded that good results of flexural tests depend more on fiber orientation than on good fiber/matrix adhesion. Flexural properties are better if the fibers are parallel within the matrix because, in such a way, oriented fibers show better resistance to the applied load [82].

**Figure 7:**
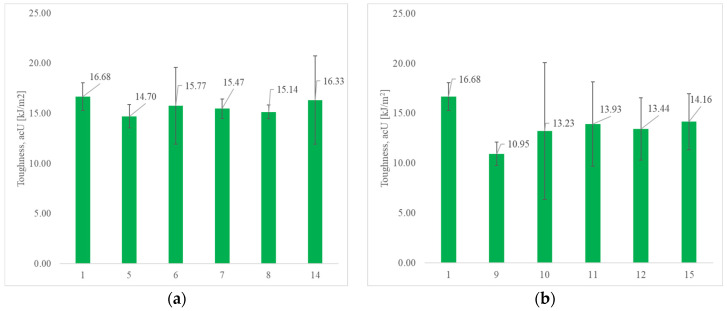
Impact properties of neat PLA compared to (**a**) SJL fiber-reinforced composites; and (**b**) SH fiber-reinforced composites. Error bars represent 95% confidence interval.

The impact properties of neat PLA and natural fiber-reinforced composites are presented in Figure 7. It can be noticed that neat PLA shows an impact strength of 16.68 kJ/m^2^ while the impact strength of other test specimens (natural fiber-reinforced composites functionalized with various fillers) decreases. These results indicate the low energy required for crack propagation, which could be influenced by weak interfacial bonding, the development of voids inside the material, and the agglomeration of fillers [83,84]. Additionally, it is important to emphasize that natural reinforcement is responsible for a higher variability of results since the coefficient of variation for natural fiber properties could range from 10 to 40% [85]; therefore, their inclusion in composites influences the higher variability of presented data.

In the case of composites reinforced with SJL fibers, a lower decrease in impact properties by 2–12% is observed, compared to composites reinforced with SH fibers by 15–35%. Test Specimens 14 and 15, in which ZnO filler is replaced with milled cork (MC), exhibit better impact properties compared to other tested composites. Upon examining Figure 7b, which illustrates the impact properties of composites reinforced with SH fibers, we observe a significant variability in the mean values of samples containing ZnO filler. Notably, if the values for Specimens 11 and 12 approach the upper limit, it can be concluded that samples reinforced with SH fibers and ZnO fillers exhibit superior impact properties. This is in line with the research conducted by [86] in which the authors confirmed the negative impact of ZnO on mechanical properties, such as flexure and tensile strength, and the positive impact on the impact strength. The presence of ZnO particles reduces the active sites and consequently, the possible bonding between the matrix and the fibers is reduced.

### 3.5. Composites Thermal Properties

**Figure 8:**
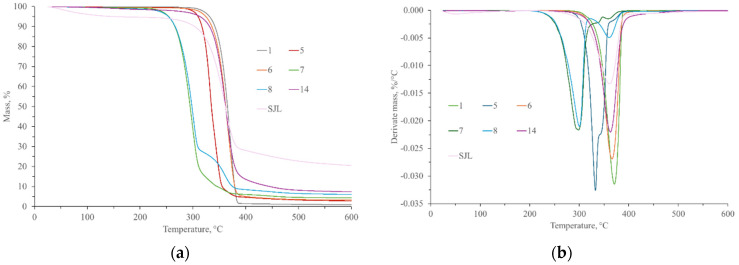
(**a**) TG and (**b**) DTG graphs of neat PLA (1), SJL fibers (SJL), and SJL composites (5–8 and 14).

**Figure 9:**
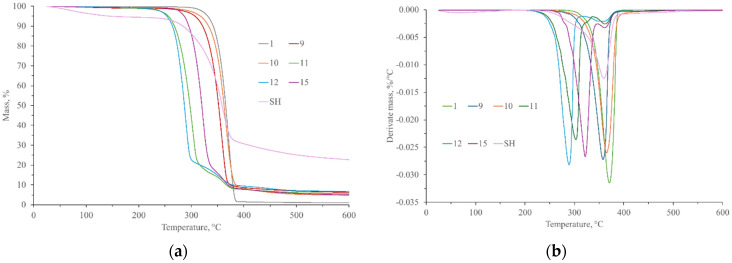
(**a**) TG and (**b**) DTG graphs of neat PLA (1), SH fibers (SH), and SH composites (9–12 and 15).

The thermal properties of composite materials were investigated through thermogravimetric analysis, differential scanning calorimetry, and microcombustion calorimetry. A thermogravimetric analysis (TG and DTG graphs) of all tested specimens is presented in Figure 8 and Figure 9. Specimen 1 (neat PLA) started to decompose at 349.98 °C. The highest weight loss (approx. 98.28%) was observed at 370.08 °C, which is ascribed to the degradation of polymeric chains [3,38,45,87].

The TG values of SJL and SH fibers refer to two degradation stages. The first stage (34 °C–103 °C) is linked to the adsorbed water loss, which is about 3.98% and 4.83% for SJL and SH fibers, respectively. The second stage (320 °C–378 °C) is attributed to the loss of the main structural constituents of the fibers (cellulose, hemicellulose, and lignin). Maximum weight loss was approx. 60% and appeared at temperatures of 352.86 °C and 360.98 °C for SJL and SH, respectively.

Composites reinforced with SJL natural fibers and without additives/fillers (Specimen 5) show a lower temperature of degradation in comparison to the neat PLA. The onset temperature of thermal degradation was 320.49 °C while the point of maximum rate of thermal degradation was 332.41 °C. Natural fiber reinforcement influences the start of thermal decomposition and shifts it to lower values [88]. The addition of linseed oil (i.e., Specimen 6) influences the improvement of thermal stability in comparison to Specimen 5 since the temperature where maximum degradation is achieved has been increased by 10.11%. Various fillers like MMT, ZnO, and MC were added to the composite material to improve the overall properties of the material (mechanical, thermal, and antibacterial). It can be observed that specimens with the ZnO filler show two degradation stages. The onset of the first degradation stage was within a temperature range of 271 °C to 276 °C while the point of maximum rate of thermal degradation was within a range of 298 °C to 300 °C and maximum weight loss was 84.68% for Specimen 7 (without MC) and 69.78% for Specimen 8 (with MC). The onset of the second degradation stage was within a temperature range of 345 °C to 353 °C while the point of the maximum rate of thermal degradation was within the range of 359 °C to 362 °C. Maximum weight loss was 4.61% for Specimen 7 (without MC) and 16.59% for Specimen 8 (with MC). Milled cork (MC) mainly consists of suberin, lignin, and cellulose [29]. Ghonjizade-Samani, F. et al. (2023) revealed that suberin, which is a complex polyester commonly found in plant cell walls enhances the thermal stability of cork [30]. Specimen 14 represents composite material without ZnO filler. Its onset temperature was 340.39 °C while the point of maximum rate of thermal degradation was 363.08 °C with a maximum weight loss of 90.38%.

Composites reinforced with SH natural fibers and without additives/fillers (Specimen 9) show a slightly higher temperature of degradation compared to SJL fiber-reinforced composites. Its point of maximum rate of thermal degradation was at 358.10 °C and maximum weight loss was 89.45%. The addition of LO (Specimen 10) increases thermal stability although the residue content after thermal analysis was slightly lower than in Specimen 9. Specimens with ZnO show a wider temperature range (288 °C–303 °C) where the point of maximum rate of thermal degradation appeared if we compared it with SJL-reinforced composites. Specimen with milled cork (Specimen 12) shows insignificantly lower overall weight loss (approx. 86.85%) and a higher residue content of 6.61% than specimen without milled cork (overall weight loss was approx. 87.06% and residue content was 5.57%). Specimen 15 is without ZnO filler, and its onset temperature of degradation was 355.05 °C while the point of maximum rate of thermal degradation was 362.11 °C, which is insignificantly lower than Specimen 14.

Based on the onset temperature during thermogravimetry analysis, safe working temperature for composite processing is determined to be in the range of 220 °C to 250 °C.

**Figure 10:**
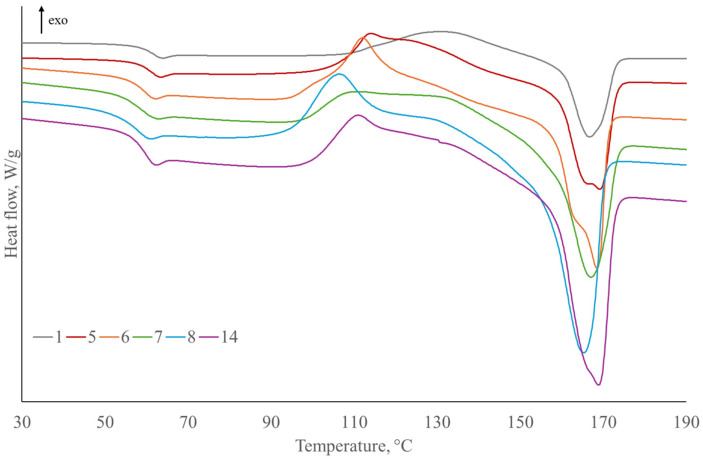
DSC curves of second heating, melting, and cold crystallization for SJL-reinforced composites compared to neat PLA.

**Figure 11:**
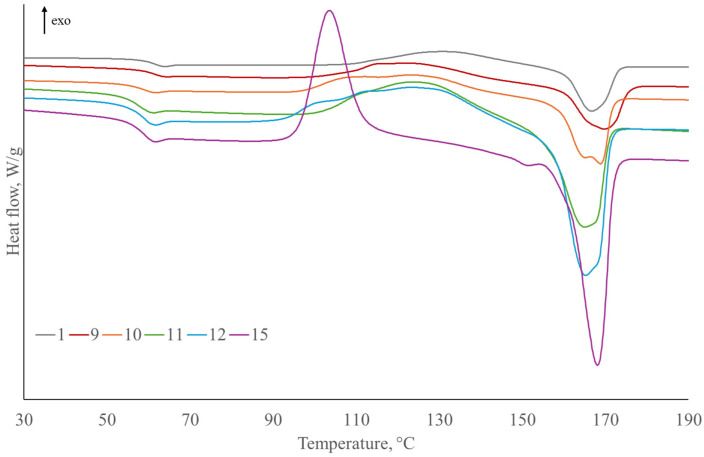
DSC curves of second heating, melting, and cold crystallization for SH-reinforced composites compared to neat PLA.

**Figure 12:**
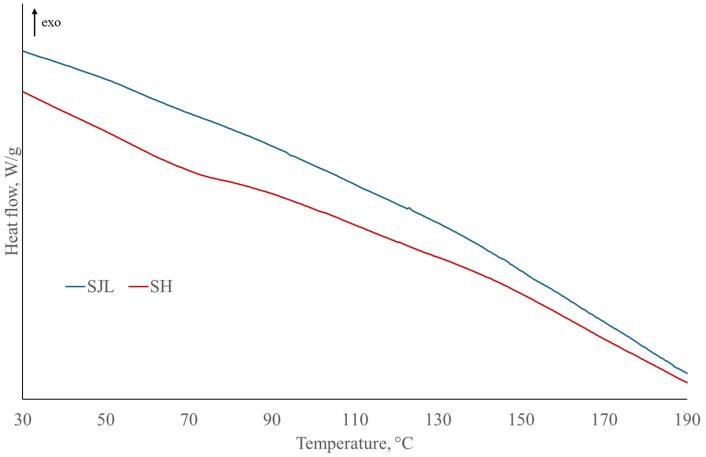
DSC curves of SJL and SH fibers.

DSC results are presented in Table 6 and Table 7 and Figure 10, Figure 11 and Figure 12 in order to determine thermal transitions of fibers and composites such as glass transition temperature, crystallization, and melting temperatures. Glass transition temperature (Tg) presents the point where an amorphous component of polymer transforms from “glassy” to “rubbery” state. It depends on the polymer type, curing process, and moisture content. Therefore, it can serve to identify the changes in the composites upon the moisture uptake, post-curing, plasticization, or even fiber–matrix debonding [89].

The glass transition temperature of composites reinforced with SJL is lower in comparison to the neat PLA (Figure 10). Lignin present in the fibers acts as a plasticizer, which lowers the glass transition [90]. Composites reinforced with SJL fibers functionalized with all four additives exhibit the largest decrease in glass transition by 3.51 °C. Linseed oil contains unsaturated fatty acids, which can interact with the ester groups in PLA, and influence the polymer’s molecular packing and chain dynamics. These interactions may cause a reduction in glass transition [91]. ZnO acts as a physical crosslinking agent and hinders the mobility of PLA chains [92]. Furthermore, MMT can also reduce the mobility of the polymer chains. On the other hand, the intercalation or exfoliation of clay layers in the PLA matrix can lead to increased molecular interactions, contributing to a higher glass transition. Applied ZnO and MMT concentration and their interactions are low and, therefore, do not have a significant influence on glass transition.

Cold crystallization temperature is significantly lower in all composites compared to neat PLA. The decrease occurred due to an increase in the chain mobility of PLA. The crystallization process was accelerated due to the increase in segmental mobility of the PLA chains by plasticization [77,93]. The melting temperature has not significantly changed. However, a second melting peak at lower temperatures appeared and is more prominent in some specimens, e.g., Specimens 5, 6, 9, and 10 (specimens without ZnO, MMT, and MC). The presence of two melting peaks on thermograms of plasticized PLA, due to the formation of crystallite with different sizes and perfection, is caused by lamellar rearrangements during PLA crystallization [94]. The degree of PLA crystallization decreased with the addition of SJL fibers indicating a dominant structure of the amorphous phase in SJL fibers.

The most prominent increase in polymer crystallinity is observed on Specimen 8 containing LO, MMT, ZnO, and cork, which indicates induced nucleation caused by the addition of cork to PLA composite [95].

The addition of SH fibers in PLA (Figure 11) increases glass transition by 0.71 °C due to higher Tg of neat SH fibers (Figure 12). The addition of LO, MMT, ZnO, and MC caused a decrease in the glass transition below Tg of neat PLA, where the most significant decrease was caused by the addition of oil. Cold crystallization temperature is lower in all SH-reinforced composites compared to neat PLA. The melting temperature of SH composites has not changed significantly, and this trend is similar to SJL-reinforced composites. The degree of crystallization of PLA increased by 2.91% with the addition of SH fibers indicating that the structure of SH fibers is more crystalline. The addition of plasticizer LO decreases crystallinity by 3.42%, which is below the crystallinity of neat PLA. On the other hand, Specimen 12 containing LO, MMT, ZnO, and MC exhibits a 15.18% degree of crystallinity indicating a positive effect of larger crystals formation on the stiffness, durability, and heat resistance of the natural fiber-reinforced polymer. The reason why there are large differences in the degree of crystallinity of pure PLA and its composites is precisely due to the increased density of nucleating sites provided by different types of fiber reinforcement and various fillers [96]. According to Da Silva, S.P.M. et al. (2021) degree of crystallinity values of cork/PLA biocomposites increased with the addition of cork indicating cork acts as a heterogeneous nucleating agent which is in line with the DSC curve of Specimen 15 presented in Figure 11. The presence of a sharp cold crystallization peak in Specimen 15 that is shifted to a lower value (103.54 °C) corresponds to a more defined crystal structure of PLA polymer stimulated by the presence of cork [97].

**Figure 13:**
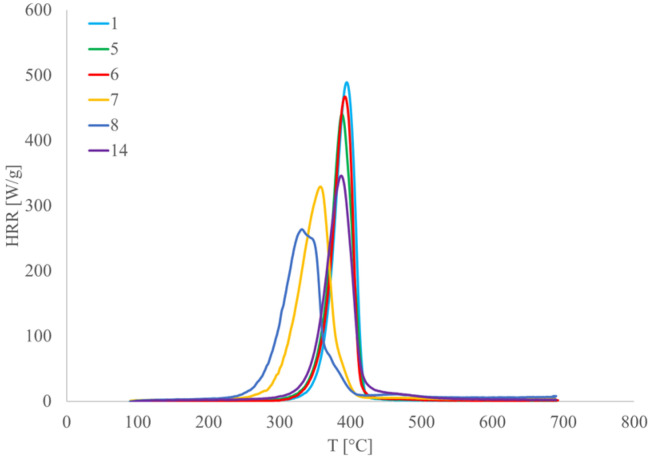
MCC results of SJL-reinforced composites.

Figure 13 presents MCC curves of SJL-reinforced composites compared to neat PLA (Specimen 1). It is noticeable that MMT-treated specimens (Specimens 7 and 8) exhibit lower heat release values than other tested specimens pointing to lower flammability of these specimens. This trend is noticed in SH-reinforced composites as well. The explanation for this trend is the formation of a char layer on the composite surface, which causes a barrier during the combustion. This barrier slows down the release of volatile gases and reduces the overall heat release rate. Therefore, the heat release rate (W/g) of Specimens 7 and 8 is 30.25% and 37.13% lower, in comparison to neat PLA. After exposure to 700 °C, only Specimens 7 and 8 have a noticeable yield of pyrolysis (2.00% for Specimen 7 and 6.67% for Specimen 8). This parameter is crucial because it indicates how much of the original material has decomposed into volatile gases versus how much remains as solid residue after the pyrolysis stage.

## 4. Conclusions

This paper presents a comprehensive overview of circular biofiber and biocomposite production, considering the zero waste concept. This concept utilizes waste from one industry (agriculture) in another industry (textile) and then repurposes waste side streams from textile production into the next industry (biofuels). Our idea to apply solid residues after the fiber isolation process (approx. 40%) to produce solid or gas biofuels (briquettes, pellets, biogas, and (bio)methane) has already been confirmed [98,99,100].

The results revealed that SH fibers isolated in the first step have more crystalline areas in their structure than SJL fibers. Due to their higher crystallinity, SH fibers exhibit 38.92% higher tensile strength values than SJL fibers. The strength of the constituents affects the final strength of the biocomposite, therefore composites reinforced with SH fibers show better results regarding tensile strength compared to SJL composites. The strength of the SH-reinforced composite has increased by 14.4% and only 2.3% for the SJL composite, compared to neat PLA. The addition of vegetable drying oil improved the interfacial properties between the fiber and the polymer. Therefore, the tensile strength is increased by 4.9% for the SJL composite and up to 22.6% for the SH composite. Young’s modulus, which indicates stiffness, is also the highest in composites with the addition of vegetable oil.

The addition of ZnO fillers has a negative influence on the impact strength of both SJL- and SH-reinforced composites, and this issue must be solved to enable composite usage for a wider range of applications, from automotive parts to construction materials. All the tested natural fiber-reinforced composites exhibit good resistance to bending making them an attractive option in industries that demand materials with good bending resistance, while also prioritizing environmental impact and cost-efficiency.

The best results of thermal stability have been observed on SH composites with MMT nanoclay, which exhibits the lowest heat release rate and clearly confirms their lower flammability and therefore, the possibility of application for flame retardant purposes.

It is important to stress that revitalization of the Spanish broom and Virginia mallow fiber production is highly desirable due to the ecological and economic benefits of its application for biocomposite materials. Namely, approximately 30% of the fibers used in the European auto industry are produced in EU member states, while 70% are imported from Eastern Europe and Asia. The composite material will be more sustainable if the raw materials used in its production are locally available.

## 5. Future Perspectives

The European Commission introduced a new Circular Economy Action Plan in 2020, which strongly aligns with the objectives of the European Green Deal: to enhance the development of cleaner and more competitive Europe. Transition to a circular economy requires changes across the entire value chain, including efficient resource management, ecodesign of sustainable products, new business and market models, innovative ways to turn waste into resources, and shifts in consumer behavior. This transformation involves a complete overhaul of the current economic model through innovation. Significant effort should be directed towards waste separation and its usage for new purposes.

The development of natural fiber-reinforced polymer composites is one of the possible ways to respond to all the requirements set by the European Green Deal for a more sustainable future. Parameters that influence the performance and properties of NFRCs are categorized into fiber-related, matrix-related, and external factors (curing process, manufacturing techniques, and environmental factors).

Fiber-related factors commonly include strength, length, content, and orientation. In this study, bast fibers of *Sida hermaphrodita* and *Spartium junceum* were used. Our results confirmed that it is worth revitalizing the local production of both plants. SH plant can be an exceptional renewable resource for biofibers and biofuel production and therefore, it is of great interest to increase its biomass yield. One possibility is introduced by Nabel, M. et al. (2018) who have suggested intercropping *Sida hermaphrodita* with legumes [15]. For this reason, our future research will be expanded to the simultaneous cultivation of *Sida hermaphrodita* and *Spartium junceum*.

Polymer matrix-related factors commonly include the type (thermoset or thermoplastic), ease of handling, and viscosity. In our future research, the polymer matrix will be revised since the poor wettability of PLA fibers resulted in the creation of voids inside the composites. Kempe, A. et al. (2015) indicated that the ratio of the breaking strain of the fibers to the breaking strain of the matrix should be at least 1:3 to obtain adequate matrix reinforcement [65]. The results presented in this paper do not correspond with this range, therefore, future research will be directed towards the modification of PLA polymer to increase its flexibility and ductility.

For enhancement of fiber/matrix interfacial properties and therefore enhancement of NFRCs mechanical and thermal properties, further chemical modifications in a more environmentally friendly manner such as milder alkali treatment, citric acid treatment, enzymatic treatment, and bio-based crosslinking will be performed.

Considering the significant loss in tensile and flexural strength of NFRC modified with ZnO, other solutions for antimicrobial functionalization will be investigated. The 1 wt.% amount of ZnO applied in this work is below the optimal concentration for efficient enhancement of mechanical and thermal properties of composite material. According to Thipperudrappa, S. et al. (2020) the optimal ZnO concentration is 2 wt.% [86]. An increase in weight/volume fractions of fillers (to more than 1 wt.%) and an increase in fiber content (up to 50–70% volume fraction of fibers) is necessary for satisfactory results, which is in line with the conclusions of other scientists [68,73].

To be able to fully close the loop of circular biocomposite production, the reuse of extracted alkaline solution for a new cycle must be performed [101]. Another solution is its evaporation and further usage of solid residue, rich in lignin, as an additive in pellet production. The applied cascade approach and zero waste concept contribute to waste minimization and make a significant contribution towards the achievement of the goals set by the European Green Deal.

## 6. Patents

Patent pending PCT/EP2023/08247 Biocomposites of Antimicrobial Properties Based on Renewable Polymers and Lignocellulosic Fibers, 16 November 2023, European Patent Office, The Hague, Inventors: Zorana Kovačević, Sandra Bischof, Tajana Krička and Nikola Bilandžija.

## Figures and Tables

**Table 1:** Specimens legend.

Specimen	Description/Constituents
Specimen 1	PLA
Specimen 5	PLA + SJL
Specimen 6	PLA + SJL + LO
Specimen 7	PLA + SJL + LO +MMT + ZnO
Specimen 8	PLA + SJL + LO +MMT + ZnO + MC
Specimen 9	PLA + SH
Specimen 10	PLA + SH + LO
Specimen 11	PLA + SH + LO + MMT + ZnO
Specimen 12	PLA + SH + LO + MMT + ZnO + MC
Specimen 14	PLA + SJL + LO +MMT + MC
Specimen 15	PLA + SH + LO + MMT + MC

Where: PLA—polylactide polymer, LO—linseed oil, MMT—montmorillonite nanoclay, ZnO—zinc oxide, MC—milled cork.

**Table 2:** Mechanical properties of SJL and SH fibers.

Fiber	Fineness (dtex)	Strain (%)	Tenacity (cN/tex)	Strength (MPa)	Young Modulus (cN/tex)	Young Modulus (GPa)
SJL	42.73 ^b^ ± 2.16	6.20 ^a^ ± 0.20	57.48 ^b^ ± 2.52	913.98 ^b^ ± 40.12	1083.93 ^b^ ± 50.33	17.24 ^b^ ± 0.80
SH	53.71 ^a^ ± 1.06	5.56 ^b^ ± 0.06	79.85 ^a^ ± 2.23	1240.38 ^a^ ± 34.57	1344.60 ^a^ ± 41.42	20.89 ^a^ ± 0.64

Results are presented as mean value within 95% confidence interval. The results were subjected to one-way analysis of variance (ANOVA), and the differences between means were compared using *t*-test (LSD) at the significance level *p* ≤ 0.05. Different letters indicate significant differences between mean values. Strength and modulus values were converted to SI units based on the approximation to circular cross-section of natural fibers.

**Table 3:** Possible mechanisms of filler interaction with polymer matrix for enhanced mechanical properties.

Mechanical Properties	Mechanism
Improved load transfer/efficient stress distribution	Interfacial bonding between fillers and matrix
Increased stiffness and strength	Additional reinforcement (fillers) restrict the mobility of polymer chains
Enhanced toughness and Impact resistance	Presence of fillers creates a more tortuous path for cracks, which requires more energy for the cracks to propagate and absorbs or dissipates energy more effectively in order to improve impact resistance

**Table 4:** Tensile properties of tested biocomposites compared to neat PLA.

Specimen	Tensile Strength (MPa)	Strain at Break (%)	Young Modulus (GPa)
Specimen 1	44.35 ^ad^ ± 5.24	6.45 ^ab^ ± 3.16	0.89 ^bh^ ± 0.24
SJL			
Specimen 5	45.37 ^a^ ± 4.40	6.72 ^a^ ± 2.23	0.88 ^c^ ± 0.13
Specimen 6	46.51 ^a^ ± 5.41	5.29 ^a^ ± 2.70	1.02 ^a^ ± 0.14
Specimen 7	19.80 ^c^ ± 9.34	4.16 ^a^ ± 1.32	0.67 ^e^ ± 0.23
Specimen 8	22.64 ^c^ ± 7.93	4.61 ^a^ ± 0.32	0.03 ^f^ ± 0.005
Specimen 14	35.56 ^b^ ± 4.81	4.11 ^a^ ± 0.76	0.81 ^d^ ± 0.40
SH			
Specimen 9	50.72 ^e^ ± 15.18	6.40 ^b^ ± 2.22	0.86 ^i^ ± 0.54
Specimen 10	54.37 ^e^ ± 1.10	4.07 ^b^ ± 0.65	1.20 ^g^ ± 0.02
Specimen 11	24.30 ^g^ ± 4.61	4.71 ^b^ ± 0.34	0.04 ^j^ ± 0.007
Specimen 12	27.64 ^g^ ± 7.11	4.85 ^b^ ± 0.57	0.04 ^j^ ± 0.02
Specimen 15	33.27 ^f^ ± 4.11	5.82 ^b^ ± 0.23	0.03 ^k^ ± 0.02

Results are presented as mean value within 95% confidence interval. The results were subjected to one-way analysis of variance (ANOVA), and the differences between means were compared using Tukey HSD–Kramer test and Q test at the significance level *p* ≤ 0.05. Different letters indicate significant differences between mean values.

**Table 5:** Flexural properties of tested biocomposites compared to neat PLA.

Specimen	Flexural Strength (MPa)	Flexural Deformation (%)	Flexural Modulus (MPa)
Specimen 1	62.83 ^bc^ ± 11.98	2.87 ^ab^ ± 0.27	2346.07 ^ek^ ± 296.18
SJL			
Specimen 5	64.34 ^bd^ ± 8.48	2.76 ^a^ ± 1.49	3554.09 ^b^ ± 608.31
Specimen 6	76.21 ^a^ ± 18.50	3.12 ^a^ ± 0.97	3471.38 ^c^ ± 434.74
Specimen 7	62.79 ^cd^ ± 13.21	2.07 ^a^ ± 0.22	3621.58 ^a^ ± 344.60
Specimen 8	55.71 ^e^ ± 18.09	2.54 ^a^ ± 0.37	3468.78 ^c^ ± 691.66
Specimen 14	68.87 ^b^ ± 8.40	2.74 ^a^ ± 0.49	3391.57 ^d^ ± 496.49
SH			
Specimen 9	101.67 ^f^ ± 19.18	2.68 ^b^ ± 0.31	4104.94 ^j^ ± 485.61
Specimen 10	86.46 ^g^ ± 9.37	2.51 ^b^ ± 0.52	4524.71 ^h^ ± 718.93
Specimen 11	68.75 ^i^ ± 13.94	1.88 ^b^ ± 0.01	4994.36 ^g^ ± 953.89
Specimen 12	79.06 ^h^ ± 1.65	2.02 ^b^ ± 0.37	5026.73 ^f^ ± 540.21
Specimen 15	74.38 ^h^ ± 16.82	2.11 ^b^ ± 0.69	4462.08 ^i^ ± 671.74

Results are presented as mean value within 95% confidence interval. The results were subjected to one-way analysis of variance (ANOVA), and the differences between means were compared using Tukey HSD–Kramer test and Q test at the significance level *p* ≤ 0.05. Different letters indicate significant differences between mean values.

**Table 6:** DSC analysis of tested biocomposites reinforced with SJL fibers, compared to neat PLA.

Phase Transition	Parameters	Specimen 1	Specimen 5	Specimen 6	Specimen 7	Specimen 8	Specimen 14	SJL
Glass transition	Tg, on (°C)	57.86	57.55	55.83	53.96	53.19	56.17	51.36
	Tg, mp (°C)	59.74	59.33	58.14	57.54	56.23	58.4	57.65
Cold crystallization	ΔHcc (Jg^−1^)	32.61	40.84	31.55	28.09	24.83	30.36	/
	Tcc, on (°C)	107.75	105.49	102.06	97.24	93.38	98.77	/
	Tcc, p (°C)	131.12	114.13	111.97	111.74	106.44	111.05	/
	Tcc, f (°C)	151.71	144.59	123.06	148.19	121.02	132.31	/
Melting	ΔHm (Jg^−1^)	34.66	41.76	35.39	31.66	31.03	31.27	/
	Tm, on (°C)	159.72	158.83	157.8	148.2	155.24	158.34	/
	Tm, p (°C)	166.35	168.65	168	166.61	164.93	168.31	/
	Tm, f (°C)	173.01	173.07	171.13	177.86	170.13	172.51	/
	Xc (%)	2.20%	1.24%	5.90%	5.65%	9.95%	1.44%	/

**Table 7:** DSC analysis of tested biocomposites reinforced with SH fibers, compared to neat PLA.

Phase Transition	Parameters	Specimen 1	Specimen 9	Specimen 10	Specimen 11	Specimen 12	Specimen 15	SH
Glass transition	Tg, on (°C)	57.86	57.92	55.96	53.81	54.66	55.07	62.75
	Tg, mp (°C)	59.74	60.47	58.34	56.38	57.03	57.51	67.86
Cold crystallization	ΔHcc (Jg^−1^)	32.61	28.04	28.56	32.64	26.33	30.1	/
	Tcc, on (°C)	107.75	104.86	119.07	100.77	98.98	96.24	/
	Tcc, p (°C)	131.12	122.75	123.23	124.08	120.74	103.54	/
	Tcc, f (°C)	151.71	140.2	143.23	146.19	143.27	111.74	/
Melting	ΔHm (Jg^−1^)	34.66	31.84	29.66	33.3	35.79	32.45	/
	Tm, on (°C)	159.72	159.15	152.43	155.59	156.66	160.49	/
	Tm, p (°C)	166.35	169.24	168.39	164.55	164.37	167.66	/
	Tm, f (°C)	173.01	175.42	171.59	170.73	170.6	171.8	/
	Xc (%)	2.20%	5.11%	1.69%	1.04%	15.18%	3.72%	/

## Data Availability

The raw data supporting the conclusions of this article will be made available by the authors on request.
